# Birthweight, parental age, birth order and breast cancer risk in African-American and white women: a population-based case–control study

**DOI:** 10.1186/bcr931

**Published:** 2004-09-22

**Authors:** M Elizabeth Hodgson, Beth Newman, Robert C Millikan

**Affiliations:** 1 Department of Epidemiology, School of Public Health, University of North Carolina at Chapel Hill, Chapel Hill, North Carolina, USA; 2 School of Public Health, Queensland University of Technology, Kelvin Grove, Queensland, Australia; 3 Department of Epidemiology, School of Public Health, and Lineberger Comprehensive Cancer Center, School of Medicine, University of North Carolina, Chapel Hill, North Carolina, USA

**Keywords:** African-American, birthweight, breast cancer, maternal age, prenatal

## Abstract

**Introduction:**

Much recent work has focused on hypotheses that very early life exposures influence adult cancer risk. For breast cancer it has been hypothesized that high *in utero *estrogen exposure may increase risk.

**Methods:**

We used data from the Carolina Breast Cancer Study, a population-based case–control study of incident breast cancer in North Carolina, to examine associations for three possible surrogates of high prenatal estrogen exposure: weight at birth, maternal age, and birth order. We also examined paternal age. Birthweight analyses were conducted for white and African-American women born in North Carolina on or after 1949 (196 cases, 167 controls). Maternal age was analyzed for US born participants younger than 49 years of age (280 cases, 236 controls).

**Results:**

There was a weak inverse association between birthweight in the highest tertile and breast cancer overall (odds ratio [OR] 0.7, 95% confidence interval [CI] 0.4–1.2), although associations differed by race (OR 0.5, 95% CI 0.2–1.0, and OR 1.0, 95% CI 0.5–2.1 for African-American and white women, respectively). For maternal age there was an approximately threefold increase in risk in women whose mothers were older than 22 years of age, relative to 19–22 years of age, when the women were born. After adjustment for maternal age, older paternal age increased risk in the oldest and youngest age categories (relative to 23–27 years of age at the woman's birth: OR 1.6, 95% CI 0.8–3.1 for age 15–22 years; OR 1.2, 95% CI 0.7–2.2 for age 28–34 years; and OR 1.5, 95% CI 0.7–3.2 for age 35–56 years). There was no association with older paternal age for white women alone. After adjustment for maternal age (265 cases, 224 controls), a birth order of fifth or higher relative to first had an inverse association with breast cancer for women younger than 49 years old (OR 0.6, 95% CI 0.3–1.3).

**Conclusion:**

Although the CIs are wide, these results lend support to the possibility that the prenatal period is important for subsequent breast cancer risk, but they do not support the estrogen hypothesis as a unifying theory for the influence of this period.

## Introduction

Recent epidemiologic studies have investigated the possibility that very early life exposures increase adult cancer risk. Trichopoulos [1] postulated that a highly estrogenic intrauterine environment would create a 'fertile soil' for carcinogenesis in breast tissue and lead to higher risk for breast cancer later in life. Because retrospective prenatal hormone measurements cannot be obtained for large numbers of people, he and others proposed that birth and maternal characteristics be investigated as surrogates for a highly estrogenic intrauterine environment. These birth characteristics include high birthweight, maternal age 20–24 years at birth, and low birth rank. Much work during the past decade has been done on birthweight in particular. There is an apparent modest positive association between high birthweight and breast cancer that is stronger in younger women, which is consistent with the estrogen hypothesis. Data on other surrogates of intrauterine estrogen levels have been less consistent [2].

Despite an overall higher incidence of breast cancer in white women, incidence and mortality rates are higher in young African-American women than in young white women [3,4]. This crossover in incidence rates, occurring at about age 40 years for women diagnosed between 1950 and 1969, was documented in the Third National Cancer Survey [4]; SEER (Surveillance, Epidemiology and End Results) data from 1997 document a shift in the crossover to approximately 45 years of age [3]. Consequently, it is important to investigate relationships between putative causes of breast cancer and breast cancer incidence in younger African-American women. To our knowledge no studies published to date have specifically addressed the relationships between prenatal or birth characteristics and breast cancer in African-American women.

The goal of this study was to characterize the relationships of birthweight, maternal age, paternal age, and birth order with breast cancer in African-American and white women in a population-based study. We analyzed data from a subset of women participating in the Carolina Breast Cancer Study (CBCS) [5], a population-based case–control study that over-sampled younger women and African-American women.

## Methods

### Study design and supplemental data collection

The CBCS (phase I) is a population-based, case–control study of incident invasive breast cancer conducted between May 1993 and December 1996 in 24 counties of central and eastern North Carolina [5]. Participants gave informed consent using forms approved by the Institutional Review Board of the University of North Carolina School of Medicine, which were in compliance with the Helsinki Declaration. Cases (*n *= 861) were women aged 18–74 years, who were mentally competent and resident in the study area at the time of selection with a first diagnosis of histologically confirmed primary invasive breast cancer. They were identified in cooperation with the North Carolina Central Cancer Registry [6] using a randomized recruitment protocol [7] to over-sample African-American women and women under 50 years of age. Potential controls were identified by North Carolina Division of Motor Vehicles (women aged 20–64 years) and/or Health Care Financing Administration (women aged 65–74 years) lists and had no previous or current history of breast cancer. Controls (*n *= 790) were frequency matched by race and 5-year age group to cases. Trained nurse interviewers collected information and obtained height and weight measurements during interviews conducted at the participant's home. To obtain birthweight and parental ages we requested birth records for all study participants born in the USA on or after 1 January 1948.

### Analytic datasets

Maternal age was analyzed in the subset of women for whom birth records with maternal age were available (Table 1) and was categorized as 15–18 years, 19–22 years, 23–27 years, or 28–44 years, based on homogeneity of risk apparent in smoothed lowess curves [8]. Paternal age was available for 92.7% of women with maternal age data, and was categorized as 15–22 years, 23–27 years, 28–34 years, or 35–56 years, by the same method. Maternal and paternal age distributions did not permit identical categorizations.

Birthweight analyses were restricted to women born in North Carolina (NC-born; Table 1). Of case and control birth records, 96% and 97%, respectively, were located and nearly all (97.0% and 94.9%, respectively) recorded birthweight. A restricted birthweight dataset was constructed that excluded women who had any of the following indicators of a possibly poorly measured birthweight: noninstitutional birth, birth attendant other than a physician, and a birthweight recorded only in pounds. Overall, birthweight was recorded only in pounds more often for African-American than for white women (28% versus 7.4%). African-American women were more likely than white women to have been born at home, were less likely to have been delivered by a physician at home, and were less likely to have had a birthweight recorded in pounds and ounces under any birth circumstances. Hence, a disproportionate number of African-American women were excluded from the restricted dataset. Birthweights were converted from pounds and ounces to grams for analysis. Race-specific tertiles were derived from white or African-American controls.

Birth order was analyzed twice, first in the full CBCS dataset and then in the subset of women for whom there was information on maternal age (Table 1). Birth order was self-reported and was a categorized as first, second to fourth, and fifth or higher.

### Statistical analysis

Unconditional logistic regression was used for all analyses. Odds ratios (ORs) and 95% confidence intervals (CIs) were the primary measure of association. PROC GENMOD in SAS (SAS Institute Inc., Cary, NC, USA) was used with an offset term to account for the age and race specific sampling probabilities used to identify eligible participants [7]. All estimates presented are adjusted for, at minimum, age and sampling fractions. Except for birthweight, parental age, and body mass index (BMI), all variables were based on self-report. Age at diagnosis (cases) or selection (controls) was categorized using the same 5-year age groups as the sampling protocol (20–24, 25–29, 30–34, 35–39, 40–44, 45–49, 50–54, 55–59, 60–64, and 65–69 years). The proportion of non-African-American participants who classified themselves as non-white was under 1%, and so, for the purposes of this study, non-African-Americans were classified as white.

Age at menarche (≤12 years or >12 years), age at first full-term pregnancy (nulliparous, <26 years, ≥26 years), lactation (nulliparous, ever breastfed, or never breastfed), parity (none, one, two, or three or more), BMI (≤25 kg/m^2 ^or >25 kg/m^2^, calculated from nurse interviewers' measurements of height and weight), first degree family history of breast cancer (positive if a mother, father, or full sibling had breast cancer), and menopausal status were considered potential confounders. Women younger than 50 years were considered postmenopausal if they had undergone natural menopause, bilateral oophorectomy, or irradiation to the ovaries. Multivariable logistic models were used to adjust for potential confounders [9]. A potential confounder was included in the model based on a >15% change in the β coefficient for any level of the birth characteristic relative to the referent in either white or African-American women. Lowess curves were generated using Stata version 7.0 (Stata Corporation, College Station, TX, USA). All other analyses were done using SAS version 8.01.

## Results

Racial distributions for analytic datasets are presented in Table 1. Overall, the proportion of African-American women was higher for the younger NC-born women (i.e. those eligible for the birthweight analysis) than for the full CBCS or for younger CBCS participants (born on or after 1 January 1948). Those eligible for the maternal and paternal age analyses were under 48 years of age at selection/diagnosis. Consequently, the proportion of postmenopausal women was much lower in this group than in the full dataset (11% versus 55%), as was mean age at menopause (controls 39.2 ± 6.1 years versus 44.4 ± 7.3 years). There were somewhat higher proportions of women with first births at age greater than 26 years, no family history of breast cancer, household income above the study median, higher educational level, and nonrural childhoods. Only minor differences emerged between the women eligible for the parental age analyses and those for whom parental age was obtained. This subgroup of women was slightly more likely to have had rural childhoods and lower education. The birthweight analysis was restricted to younger NC-born women. These women reported, on average, only slightly lower educational level, more rural childhoods, lower household income, lower age at first birth, and higher BMI than did younger women overall.

No important differences in breast cancer risk factors emerged between those eligible for the birthweight study and those for whom birthweight was obtained. ORs for age at menarche, age at first pregnancy, and lactation were virtually identical in the full CBCS and all analytic datasets. Differences in ORs between the full CBCS dataset and the birthweight dataset were, as expected, due to age restriction in the latter. In the birthweight dataset, ORs for family history were slightly higher (OR 1.6, 95% CI 1.0–2.7 versus OR 1.4, 95% CI 1.0–1.9), whereas ORs for the following were slightly lower: BMI greater than 25 kg/m^2 ^(OR 0.6, 95% CI 0.4–0.8 versus OR 0.8, 95% CI 0.6–1.0), postmenopausal status (OR 0.7, 95% CI 0.4–1.5 versus OR 0.9, 95% CI 0.7–1.2), and parity of three or greater (OR 0.5, 95% CI 0.2–1.1 versus OR 0.8, 95% CI 0.6–1.1).

### Risk factor distributions

The distributions of birthweight, parental age, and birth order are presented in Table 2. Birth records were unavailable from some states, increasing the proportion of NC-born women in the maternal age dataset. The birthweight dataset was restricted to NC-born participants because of unavailability of birthweight on most out-of-state birth records. Ages at diagnosis/selection were similar in analytic datasets and relevant subgroups of CBCS cases and controls. Maternal age, paternal age, and birth order were also similarly distributed in datasets including only younger women. As expected, the frequency of birth orders higher than fourth was lower among the younger women.

### Birthweight

Tables 3 and 4 present ORs and 95% CIs for the association between birthweight categories and breast cancer in the full and restricted datasets, combined and by race, respectively. Overall, there was a weak inverse association between higher birthweight and breast cancer in the full dataset but not in the restricted dataset. Higher birthweight was inversely associated with breast cancer among African-American women in the full and restricted datasets, although CIs were wide. There was no association for higher birthweight in white women for the full dataset and a modest but statistically nonsignificant positive association for the restricted dataset. There was no association between lower birthweight and breast cancer for white or African-American women in the full birthweight dataset. As has historically been the case in North Carolina and elsewhere [10], mean and median birthweight and lower and upper limits of birthweight distributions among controls were higher for whites than for African-Americans. Neither prenatal characteristics nor adult BMI were strongly correlated with birthweight (data not shown).

### Maternal and paternal age

ORs for maternal and paternal age and breast cancer are presented in Table 5. After adjustment, older maternal age (>22 years of age) increased ORs approximately threefold, whereas older paternal age (>27 years of age) was more weakly associated with breast cancer. Maternal and paternal ages, as categorized, were moderately correlated (Spearman correlation coefficient 0.73, 95% CI 0.69–0.78). Parental ages were somewhat correlated with birth order (Spearman correlation coefficients 0.47, 95% CI 0.40–0.54 and 0.43, 95% CI 0.35–0.50 for maternal and paternal ages, respectively). After full adjustment, the OR was elevated and of borderline statistical significance for maternal age 15–18 years among African-American women but not among white women (Table 6). ORs for maternal age over 22 years were increased twofold to fivefold, with 95% CIs usually excluding the null, for both white and African-American women. The odds of breast cancer for all categories of maternal age were slightly stronger for first-born participants, although this was not statistically significant (data not shown).

After adjustment for maternal age, birth order, adult BMI, and household income, there was no association between paternal age and breast cancer among white women. For African-American women, ORs were elevated for both younger (15–22 years of age) and older (35–56 years of age) paternal ages, although CIs were wide. There was no substantial difference in results when the maternal/paternal age datasets were restricted to women born on or after 1 January 1949. Among controls, parental ages were distributed similarly by race, with African-American participants having slightly higher mean maternal and paternal ages than whites.

### Birth order

Results for analyses of birth order are shown in Tables 7 and 8. In the full CBCS dataset, birth order (categorized as first, second to fourth, or fifth or higher) was not associated with breast cancer, overall or by race. In the full CBCS, mean birth order was higher for African-Americans than for whites; this pattern was stronger in younger CBCS participants. No potential confounders met the criteria for model inclusion. Finer categorization of birth order did not change the results. For younger women, adjustment for maternal age, adult BMI, and household income revealed a weak, statistically nonsignificant, inverse relationship between higher birth order and odds of breast cancer, which did not differ appreciably by race.

## Discussion

We examined the relationships between breast cancer and birthweight, parental age, and birth order among women younger than 49 years of age residing in North Carolina. Overall, there was a weak inverse association with higher birthweight, which was stronger in the full dataset than in the restricted dataset. For white women in the study there was no overall association between birthweight and breast cancer. Among white women born in medical facilities, birthweight in the highest tertile was positively associated with breast cancer, but CIs were wide and included the null. Higher birthweight was inversely associated with breast cancer for African-American women regardless of delivery setting, but again CIs were wide for all estimates. Most previous studies have reported weak to modest positive associations between higher birthweight and breast cancer [11-22], with some showing a positive dose response [11,16-19]. The only previous report of an overall weak inverse association between higher birthweight and breast cancer was from an Asian population [23], although similar results were reported among older, white women [16,19]. Two studies have shown no association [24,25]. With the exception of the Asian case–control study [23], previous studies of birthweight have been done in exclusively or predominantly white populations: seven large record-based, nested case–control studies in Scandinavian cohorts [11,12,14,16,17,22,24]; five population-based, case–control studies in the USA [13,19,20,25,26]; one US cohort study [18]; and one cohort study [21] and a cross-sectional study [15] conducted in the UK. All previous studies of birthweight and breast cancer included younger women; 10 presented results for premenopausal or younger women separately [13,14,16-21,23,26]. Associations were generally positive in younger women, with ORs ranging from 1.25 (95% CI 1.0–1.6) [17] to 3.5 (95% CI 1.3–9.4) [16] for birthweights of 4000 g or greater, as compared with the ORs of 1.4–1.5 for birthweight greater than 3458 g among younger white women with reliable birthweights observed in the present study. No previous studies have estimated ORs for African-American women.

In the USA maternal report and self-report have been the most common sources of birthweight information, rather than birth records. Andersson and coworkers [27] conducted an analysis of agreement between self-reported birthweight and birth records. They found that, despite good overall agreement (Spearman correlation coefficient 0.76), 31% of self-reported birthweights differed from birth record data by 500 g or more, and that this level of misclassification led to both underestimation and overestimation of the magnitude and significance of various effect estimates. Moreover, nonresponse can be sizable (ranging from 12% [19] to 24% [28]) and may reflect bias toward healthier [29], more educated, and/or more communicative mothers. In the USA, birthweight was only routinely recorded on birth records of younger women, the group in which the association between birthweight and breast cancer appears to be strongest [2]. The two US studies that used birth records (conducted in Hawaii [26] and New York state [13]) employed a design similar to that of the present study – a population-based, case–control study using cases born in the state where they were recruited. Both observed minimal, statistically nonsignificant, increased risks for breast cancer among women in the highest tertile of birthweight relative to those in the central tertile. The present study is intermediate in sample size between these two studies, which included 74 and 484 cases, respectively.

In addition to using birth records to decrease misclassification, we performed a separate birthweight analysis for the subset of women who were delivered by physicians in hospitals or doctors' clinics and had their birthweight recorded in pounds and ounces. During the 1950s, home birth and delivery by lay midwives was common practice in North Carolina, particularly among African-Americans and in rural areas, and this could have affected data collection [10]. Additionally, participants delivered in a medical setting comprise a subgroup of women more closely comparable to previous study participants than do women born at home. Results for white women from this subset were in agreement with previous literature, with a small positive association between birthweight and breast cancer, whereas birthweight remained inversely associated for African-American women in the restricted dataset with comparable delivery circumstances. Although analyses in this restricted group potentially reduce birthweight misclassification, results may have limited generalizability to less medically advantaged populations.

Although the search strategy employed in this study limited nonlocatable birth records to 3.6% of the study population, and records missing birthweight to 4.0% of locatable records (i.e. data available for 92% and 93% of eligible cases and controls, respectively), the missing records were predominantly those of African-American women who self-reported birth in the more rural counties of North Carolina.

The inverse associations between higher birthweight and breast cancer seen in this study could also partly be explained by selection bias in the full CBCS dataset if either the case group under-represented the proportion of high birthweight women in the underlying case population or the control group under-represented the proportion of normal weight births in the underlying population. Because birthweights in North Carolina have been increasing over time, more strongly in whites than in African Americans [30], younger white women would be expected on average to have the highest birthweights, and this group is slightly over-represented rather than under-represented in the case population. Some under-representation of African-American women in the control population (36.5% response rate for younger African-Americans) could have contributed to an upward bias in the control birthweights.

In the context of a relatively disadvantaged population such as this one, a higher birthweight may be a surrogate for a different constellation of prenatal and postnatal influences than in a relatively advantaged population. Rather than viewing birthweight solely as an indicator of a highly estrogenic prenatal environment, or even specific physiologic processes, birthweight can also be viewed more globally as an indicator of the prepregnancy health of the mother [31,32]. In this context, it could be considered predictive of the general overall health of the daughter as well and perhaps of a decreased susceptibility to some etiologic agents. Socioeconomic status, based on study participants' self-reported current household income, was not found to be a confounder in this study, but it is probably a poor surrogate for complex environmental influences such as early diet, physical activity, or childhood residence. If there is either a general or breast cancer specific survival advantage to having a higher birthweight, then one would expect to see an inverse association between birthweight and breast cancer among older women. This was found in one study of birthweight and breast cancer [19] but not in another [18], although an apparent protective effect of higher birthweight has been found for other chronic diseases [19,33]. Inasmuch as birthweight is a good surrogate for higher intrauterine estrogen levels, these data do not support the hypothesis that *in utero *estrogen exposure increases risk for breast cancer.

One limitation of our birthweight analysis is that, lacking an accurate measure of gestational age, it is not possible to interpret fully the association between lower birthweight and breast cancer. Although Andersson and coworkers [11] reported increases in risk associated with birthweight after adjustment for gestational age, particularly after additional adjustment for age at menarche, evidence is inconsistent for gestational age as a strong confounder [2,14]. In the birthweight analyses, power (the probability of correctly rejecting the null hypothesis) to detect an OR of 1.5 at the 95% confidence level was low for whites and African-Americans (0.33 and 0.61, respectively). Similarly, power to detect an OR of 0.5 was low for whites and African-Americans (0.25 and 0.50, respectively). Therefore chance cannot be ruled out as an explanation for the results. Although stratification allowed us to characterize the relationships between these early life factors and breast cancer in African-American women, power to detect an overall effect was decreased. Because this is the only study to date that presents data on African-American women, further research should be undertaken.

Older maternal age exhibited a moderate positive association with breast cancer in this study. Study participants whose mothers were aged 23 years or older at the participant's birth had approximately twofold to fourfold higher odds of breast cancer than did women whose mothers were between 19 and 22 years of age. Although African-American women whose mothers were aged under 19 years also had elevated odds of breast cancer, the pattern did not differ appreciably between white and African-American women. Although the magnitude of OR for women whose mothers were aged 23–27 years was greater than in previous studies, the findings were consistent with the majority of previous reports: weak positive associations with older maternal age [13,24-26,34-42], with stronger associations (approximate doubling in the oldest categories) found for younger women [13,26,43]. Several studies, however, reported no association with older maternal age [12,17,19,43,44]. Innes and coworkers [13] reported a similar J-shaped relationship between maternal age and breast cancer for women diagnosed before age 33 years; the lowest risk was for those aged 20–24 years, with an approximate doubling of odds for women whose mothers were older than 35 years old at their birth. Collectively, these data do not support highest risk being associated with maternal age 20–24 years, as predicted by the estrogen theory [1]. Recent evidence, however, indicates that the association between maternal age and levels of pregnancy estrogens may be weaker than was previously thought [45,46]. Alternatively, older oocytes may have sustained more genetic damage over time and/or DNA repair may be deficient in older mothers [35,47].

The pattern of association between having an older father (older paternal age) and breast cancer was somewhat different for white and African-American women. After adjustment for maternal age and birth order, paternal age was not associated with breast cancer for white women. This is consistent with previous reports for white women showing little to no effect of paternal age [34,38,41,42]. For African-American women in the present study, positive associations with breast cancer were seen for those with the youngest (age 15–22 years) and oldest (age 35–56 years) fathers at their birth, even after adjustment for maternal age and birth order. This is broadly consistent with the only previous study of paternal age to include African-Americans (10.1 % of participants, 52 matched case–control pairs) [13]. In that study Innes and coworkers found an elevated risk associated with older paternal age, after adjustment for maternal age and birth order (OR 1.3, 95% CI 0.9–1.7 for age 30–34 years; OR 1.2, 95% CI 0.8–1.7 for age 35–39 years; and OR 1.3, 95% CI 0.8–2.0 for age ≥40 years), and there was some suggestion of effect modification by race. Although speculative, the differing risk patterns for paternal age in white and African-American women may reflect differing exposures for white and African-American men at that time and place, which could have affected mutation rates.

Birth records with parental age could only be obtained for 76% of CBCS participants born in or after 1948, and were missing almost exclusively by participants' state of birth. Breast cancer mortality is generally higher in the midwest and northeast regions of the USA than in the southeast [48], and so if participants with higher birthweights from those areas were systematically excluded then bias toward the null would be expected. However, there was no regional pattern to the missing birth records; therefore, this was unlikely to have introduced substantial bias. Paternal information was collected only when the mother was married. Although the proportion of unmarried parents is small, this could have introduced bias.

In women younger than 50 years of age, higher birth order exhibited a weak inverse association with breast cancer only after adjustment for maternal age. No association was seen in the full CBCS, which included women aged up to 74 years, although data were not available to adjust for maternal age. This is consistent with the majority of previous studies, which have shown either a weak inverse association with breast cancer [19,26,36,49] or no association [22,24,37]. A weak positive relationship (OR 1.05, 95% CI 1.01–1.10 per 1 unit increase in birth order) was found by Hemminki and Mutanen [50]. Few studies were able to adjust for maternal age. Because pregnancy estrogens appear to be highest in first pregnancies and decline in successive pregnancies [51,52], these results lend some support to the theory that prenatal estrogen exposure may influence breast cancer later in life. Birth order was self-reported and may have been misclassified. Although the number of previous maternal pregnancies could be a better measure of prenatal estrogen exposure than live birth order, we could not assess this because of poor data quality on the birth records.

Younger African-American women are at higher risk for breast cancer than younger white women [3,4]. Birthweight, patterns of parental age at birth, and birth order continue to vary by race [53]. Our study has several important strengths. Use of birth records as the source of birthweight information improved accuracy of the exposure measurement, eliminated recall bias caused by self-report, and reduced possible selection bias from maternal report. Similarly, birth records improved data quality for parental age. Using a population-based case–control study made it possible to evaluate a wider range of adult-life risk factors as potential confounders and/or effect modifiers than is generally possible in a registry-based study.

## Conclusion

Taken as a whole, the results for birthweight, parental age, and birth order from the present study do not support the estrogen exposure hypothesis as a unifying theory for prenatal influence on adult breast cancer. This emphasizes the importance of further investigating the influence of prenatal factors on breast cancer risk, particularly in multiple populations. Additional hypotheses must be pursued, including the association between birthweight and other hormonal exposures such as insulin-like growth factor I, and between maternal age and endogenous and exogenous mutagenic exposures. Methodologic difficulties involved in investigating prenatal exposures in nonwhite and/or disadvantaged populations are not trivial; nonetheless, this type of investigation must be done to fully understand life course processes that can culminate in breast cancer among women of any background.

## Author contributions

MEH, BN and RCM participated in the interpretation of results and writing of the manuscript. MEH performed data collection, data entry, and statistical analyses.

## Competing interests

The authors declare that they have no competing interests.

## Abbreviations

BMI = body mass index; CBCS = Carolina Breast Cancer Study; CI = confidence interval; OR = odds ratio.

## Figures and Tables

**Table 1:** Cases and controls in Carolina Breast Cancer Study (CBCS) analytic datasets by race

		African-American		White	
		
Analytic dataset	Main exposure	Cases (%)	Controls (%)	Cases (%)	Controls (%)
CBCS, entire	Birth order	335 (100.0)	332 (100.0)	526 (100.0)	458 (100.0)
CBCS, born 1948 or later		131 (39.1)	135 (40.7)	235 (44.7)	181 (39.5)
Maternal age dataset	Birth order, Maternal age	107 (31.9)	116 (34.9)	173 (32.9)	121 (26.4)
Paternal age dataset	Paternal age	95 (28.4)	100 (30.1)	171 (32.5)	118 (25.8)
CBCS, NC born 1949 or later		99 (29.6)	96 (28.9)	112 (21.3)	85 (18.6)
Birthweight, full dataset	Birthweight	86 (25.7)	89 (26.8)	110 (20.9)	78 (17.0)
Birthweight, restricted dataset	Birthweight	49 (14.6)	37 (11.1)	98 (18.6)	63 (13.8)

**Table 2:** Prenatal Factors in Carolina Breast Cancer Study (CBCS) Analytic Datasets

	CBCS								Analytic subgroups							
		
	CBCS, full dataset				CBCS, born 1948+				Maternal age dataset				Birthweight dataset			
				
	Cases		Controls		Cases		Controls		Cases		Controls		Cases		Controls	
	*n*	(%)	*n*	(%)	*n*	(%)	*n*	(%)	*n*	(%)	*n*	(%)	*n*	(%)	*n*	(%)
								
**Number**	861		790		366		316		280		237		196		167	
**Age at selection/diagnosis (years)**																
Mean ± SD	50.26 ± 11.93		51.83 ± 11.65		39.59 ± 5.29		40.65 ± 4.72		39.19 ± 5.43		40.68 ± 4.63		38.39 ± 5.29		39.95 ± 4.32	
Median	48		49		41		42		40		42		39		41	
Range	23–74		21–74		23–48		21–47		23–48		26–47		23–47		26–47	
**Birthplace**																
NC born	583	(67.7)	550	(69.6)	235	(64.2)	207	(65.5)	220	(78.6)	198	(83.5)	196	(100.0)	167	(100.0)
US born (outside NC)	261	(30.3)	218	(27.6)	121	(33.1)	98	(31.0)	60	(21.4)	39	(16.5)	0	(0.0)	0	(0.0)
Non-US born	17	(2.0)	22	(2.8)	10	(2.7)	11	(3.5)	0	(0.0)	0	(0.0)	0	(0.0)	0	(0.0)
**Prenatal factors**																
Birthweight																
Lower tertile	NA		NA		NA		NA		NA		NA		73	(37.2)	57	(34.1)
Central tertile													70	(35.7)	54	(32.3)
Upper tertile													53	(27.0)	56	(33.5)
Mean ± SD													3210.7 ± 526.1		3262.5 ± 550.3	
Maternal age (years)																
15–18	NA		NA		NA		NA		31	(11.1)	31	(13.1)	21	(10.7)	22	(13.3)
19–22									51	(18.2)	80	(33.8)	34	(17.3)	53	(31.9)
23–27									87	(31.1)	49	(20.7)	60	(30.6)	38	(22.9)
28–44									111	(39.6)	77	(32.5)	81	(41.3)	53	(31.9)
Mean ± SD									26.46 ± 6.48		25.18 ± 6.66		26.63 ± 6.46)		25.34 ± 6.82	
Paternal age (years)																
15–22	NA		NA		NA		NA		39	(14.7)	35	(16.1)	26	(14.1)	21	(14.0)
23–27									73	(27.5)	69	(31.8)	47	(25.4)	50	(33.3)
28–34									87	(32.8)	67	(30.9)	66	(35.7)	49	(32.7)
35–56									66	(24.9)	46	(21.2)	46	(24.9)	30	(20.0)
Mean ± SD									29.97 ± 7.10		29.54 ± 7.46		30.24 ± 7.06)		29.71 ± 7.48	
Birth order (self-report)																
First	297	(34.8)	268	(34.1)	117	(32.3)	108	(34.3)	88	(31.5)	80	(33.9)	56	(28.6)	53	(31.9)
Second-fourth	406	(47.5)	377	(48.0)	194	(53.6)	158	(50.2)	149	(53.4)	115	(48.7)	107	(54.6)	81	(48.8)
Fifth or higher	151	(17.7)	140	(17.8)	51	(14.1)	49	(15.6)	42	(15.1)	41	(17.4)	33	(16.8)	32	(19.3)
Mean ± SD	2.86 ± 2.30		2.87 ± 2.32		2.76 ± 2.12		2.74 ± 2.15		2.83 ± 2.22		2.84 ± 2.25		2.95 ± 2.22		3.01 ± 2.35	

NA, not available

**Table 3:** Birthweight distributions and odds ratios for breast cancer in African-American and white women combined

	Minimally adjusted OR^a^				Fully adjusted OR^b^			
		
	Cases	Controls	OR^a^	95% CI	Cases	Controls	OR^b^	95% CI
Full birthweight dataset	*n *= 196	*n *= 167			*n *= 191	*n *= 161		
Lower tertile^c^	73	57	0.9	0.6–1.6	72	55	1.0	0.6–1.7
Central tertile	70	54	Ref.		69	51	Ref.	
Upper tertile	53	56	0.7	0.4–1.2	50	55	0.7	0.4–1.2
Mean ± SD (g)		3262 ± 550						
Median (g)		3232						
Range (g)		1021–4621						
Restricted dataset^d^	*n *= 147	*n *= 100			*n *= 143	*n *= 97		
Lower tertile^c^	59	43	0.9	0.5–1.7	58	41	1.0	0.5–0.9
Central tertile	48	32	Ref.		47	31	Ref.	
Upper tertile	40	25	1.0	0.5–2.1	38	25	0.9	0.4–1.9
Mean ± SD (g)		3210 ± 482						
Median (g)		3232						
Range (g)		2041–4621						

^a^Adjusted for age, race and sampling fractions. ^b^Adjusted for age, race, sampling fractions, history of previous biopsy, maternal age, and adult body mass index >25 kg/m^2^. ^c^Tertiles are race specific with cutpoints derived from controls. White women: <3062 g, 3062–3458 g, >3458 g; African American women: <3146 g, 3146–3486 g, >3486 g. ^d^Birthweight measured in pounds *and *ounces and participant delivered in a medical facility by a physician. CI, confidence interval; OR, odds ratio.

**Table 4:** Birthweight distributions and odds ratios for breast cancer among African-American and white women by race

	Minimally adjusted OR^a^								Fully adjusted OR^b^							
		
	African-American				White				African-American				White			
				
	Cases	Controls	OR^a^	95% CI	Cases	Controls	OR^a^	95% CI	Cases	Controls	OR^b^	95% CI	Cases	Controls	OR^b^	95% CI
Full birthweight dataset	*n *= 86	*n *= 89			*n *= 110	*n *= 78			*n *= 83	*n *= 85			*n *= 108	*n *= 76		
Lower tertile^c^	38	30	1.0	0.5–2.1	35	27	0.9	0.4–1.8	38	29	1.1	0.5–2.2	34	26	0.9	0.4–2.0
Central tertile	33	29	Ref.		37	25	Ref.		32	27	Ref.		37	24	Ref.	
Upper tertile	15	30	0.5	0.2–1.0	38	26	1.0	0.5–2.1	13	29	0.4	0.2–1.0	37	26	0.9	0.4–2.0
Mean ± SD (g)		3251 ± 584				3276 ± 513										
Median (g)		3211				3239										
Range (g)		1021–4536				1843–4734										
Restricted dataset^d^	*n *= 49	*n *= 37			*n *= 98	*n *= 63			*n *= 47	*n *= 36			*n *= 96	*n *= 61		
Lower tertile^c^	26	19	0.7	0.3–2.0	33	24	1.0	0.4–2.1	26	18	0.8	0.3–2.4	32	23	1.1	0.5–2.4
Central tertile	17	9	Ref.		31	23	Ref.		16	9	Ref.		31	22	Ref.	
Upper tertile	6	9	0.4	0.1–1.4	34	16	1.5	0.7–3.4	5	9	0.3	0.1–1.2	33	16	1.4	0.6–3.2
Mean ± SD (g)		3184 ± 463)				3225 ± 497)										
Median (g)		3147				3232										
Range (g)		2296–4337				2041–4621										

^a^Adjusted for age and sampling fractions. ^b^Adjusted for age, sampling fractions, history of previous biopsy, maternal age, and adult body mass index >25 kg/m^2^. ^c^Tertiles are race specific with cutpoints derived from controls. White women: <3062 g, 3062–3458 g, >3458 g; African American women: <3146 g, 3146–3486 g, >3486 g. ^d^Birthweight measured in pounds *and *ounces and participant delivered in a medical facility by a physician. CI, confidence interval; OR, odds ratio.

**Table 5:** Parental age distributions and odds ratios for breast cancer among African-American and white women combined

	Minimally adjusted OR^a^				Fully adjusted OR^b^			
		
	Case	Control	OR	95% CI	Case	Control	OR	95% CI
		
Maternal age (years)^c^	*n *= 280	*n *= 236			*n *= 263	*n *= 235		
15–18	31	31	1.8	0.9–3.4	30	29	1.8	0.9–3.5
19–22 (ref.)	51	80	Ref.		48	77	Ref.	
23–27	87	49	3.0	1.8–5.0	85	48	3.5	2.0–5.9
28–44	111	77	2.5	1.6–4.0	100	81	3.0	1.8–5.2
Mean ± SD	26.5 ± 6.5	25.2 ± 6.7						
Median	26	23						
Range	17–43	15–43						
Paternal age (years)^d^	*n *= 266	*n *= 218			*n *= 251	*n *= 206		
15–22	39	35	1.0	0.6–1.8	38	33	1.6	0.8–3.1
23–27 (refs)	73	70	Ref		69	65	Ref	
28–34	87	67	1.3	0.8–2.1	83	62	1.2	0.7–2.2
35–56	67	46	1.6	0.9–2.6	61	46	1.5	0.7–3.2
Mean ± SD	30 ± 7.1)	29.5 ± 7.5)						
Median	29	28						
Range	17–53	15–56						

^a^Adjusted for age, race, and sampling fractions. ^b^Adjusted for age, race, sampling fractions, and other covariates as specified. ^c^Additional covariates for fully adjusted OR: household income ≥$30,000 and birth order.^d^Additional covariates for fully adjusted OR: adult body mass index >25 kg/m^2^, household income ≥$30,000, birth order, and maternal age.

**Table 6:** Parental age distributions and odds ratios for breast cancer among African-American and white women by race

	Minimally adjusted OR^a^								Fully adjusted OR^b^							
		
	African-American				White				African-American				White			
				
	Case	Control	OR	95% CI	Case	Control	OR	95% CI	Case	Control	OR	95% CI	Case	Control	OR	95% CI
				
Maternal age (years)^c^	*n *= 107	*n *= 116			*n *= 173	*n *= 120			*n *= 99	*n *= 118			n = 164	n = 117		
15–18	19	17	2.3	0.9–5.5	12	14	1.1	0.5–2.9	18	15	2.7	1.0–7.0	12	14	1.0	0.4–2.7
19–22 (ref.)	18	33	Ref.		33	47	Ref.		17	33	Ref.		31	44	Ref.	
23–27	28	28	1.9	0.9–4.3	59	21	4.2	2.1–8.3	27	28	2.4	1.0–5.4	58	20	4.8	2.3–9.8
28–44	42	38	2.2	1.1–4.6	69	39	2.7	1.5–4.9	37	42	3.3	1.4–7.4	63	39	2.8	1.4–5.4
Mean ± SD	26.4 ± 7.3	25.5 ± 7.0			26.5 ± 5.9	24.9 ± 6.3										
Median	26	23			26	22										
Range	15–43	16–38			16–43	15–44										
Paternal age (years)^d^	*n *= 95	*n *= 100			*n *= 171	*n *= 118			*n *= 89	*n *= 94			n = 162	n = 112		
15–22	16	15	1.6	0.6–3.9	23	20	0.7	0.3–1.5	16	14	2.7	1.0–7.3	22	19	1.1	0.5–2.5
23–27 (ref.)	22	30	Ref.		51	40	Ref.		20	27	Ref.		49	38	Ref.	
28–34	23	28	1.3	0.6–2.8	64	39	1.3	0.8–2.4	22	26	1.3	0.5–3.4	61	36	1.2	0.6–2.4
35–56	34	27	1.9	0.9–4.0	33	19	1.4	0.7–2.8	31	27	2.1	0.8–5.8	30	19	1.1	0.4–2.9
Mean ± SD	30.9 ± 6	30.4 ± 8.0			29.5 ± 6.8	28.8 ± 6.9										
Median	30	29			29	27										
Range	18–52	19–54			17–53	15–56										

^a^Adjusted for age and sampling fractions. ^b^Adjusted for age, sampling fractions, and other covariates as specified. ^c^Additional covariates for fully adjusted OR: household income ≥$30,000 and birth order. ^d^Additional covariates for fully adjusted OR: adult body mass index >25 kg/m^2^, household income ≥$30,000, birth order, and maternal age.

**Table 7:** Birth order distributions and odds ratios for breast cancer among white and African-American women combined

	Minimally adjusted OR^a^				Fully adjusted OR^b^			
		
	Case	Control	OR	95% CI	Case	Control	OR	95% CI
		
Full CBCS^c^	*n *= 854	*n *= 785			*n *= 854	*n *= 785		
1st born (ref.)	297	268	Ref.		297	268	Ref.	
2nd–4th born	406	377	0.9	0.7–1.1	406	377	0.9	0.7–1.1
5th–14th born	151	140	1.0	0.8–1.3	151	140	1.0	0.8–1.3
Mean ± SD	2.9 ± 2.3	2.9 ± 2.3						
Median	2.0	2.0						
Range	1–14	1–14						
Born ≥1948^d,e^	*n *= 362	*n *= 315			*n *= 164	*n *= 224		
1st born (ref.)	117	108	Ref.		84	76	Ref.	
2nd–4th born	194	158	1.1	0.8–1.6	144	109	0.9	0.6–1.4
≥5th born	51	49	1.0	0.6–1.7	37	39	0.6	0.3–1.3
Mean ± SD	2.8 ± 2.1	2.7 ± 1.2			2.8 ± 2.2	2.9 ± 2.3		
Median	2.0	2.0			2.0	2.0		
Range	1–14	1–14			1–14	1–14		

^1^Adjusted for age, race, and sampling fractions. ^2^Adjusted for age, race, sampling fractions, and other covariates as specified. ^3^Additional covariates for fully adjusted OR: none. ^4^Additional covariates for fully adjusted OR: adult body mass index >25 kg/m^2^, household income ≥$30,000 and maternal age.^5^Restricted to participants born on or after 1 January 1948. CBCS, Carolina Breast Cancer Study; CI, confidence interval; OR, odds ratio.

**Table 8:** Birth order distributions and odds ratios for breast cancer among white and African-American women by race

	Minimally adjusted OR^a^								Fully adjusted OR^b^							
		
	African-American				White				African-American				White			
				
	Case	Control	OR	95% CI	Case	Control	OR	95% CI	Case	Control	OR	95% CI	Case	Control	OR	95% CI
				
Full CBCS^c^	*n *= 331	*n *= 329			*n *= 523	*n *= 456			*n *= 331	*n *= 329			n = 523	n = 456		
1st born (ref.)	106	103	Ref.		191	165	Ref.		106	103	Ref.		191	165	Ref.	
2nd–4th born	142	151	0.8	0.6–1.2	264	226	1.0	0.7–1.3	142	151	0.8	0.6–1.2	264	226	1.0	0.7–1.3
≥5th born	83	75	1.0	0.7–1.5	68	65	1.0	0.7–1.5	83	75	1.0	0.7–1.5	68	65	1.0	0.7–1.5
Mean ± SD	3.6 ± 2.7	3.3 ± 2.7			2.6 ± 1.9	2.6 ± 2.0										
Median	2.0	3.3			2.0	2.0										
Range	1–14	1–14			1–12	1–11										
Born ≥1948^d,e^	*n *= 129	*n *= 134			*n *= 233	*n *= 181			*n *= 101	*n *= 109			n = 164	n = 115		
1st born (ref.)	37	41	Ref.		80	67	Ref.		29	31	Ref.		55	45	Ref.	
2nd–4th born	56	61	1.0	0.5–1.7	138	97	1.2	0.8–1.9	46	50	0.7	0.4–1.5	98	59	1.0	0.6–1.8
≥5th born	36	32	1.2	0.6–2.3	15	17	0.7	0.3–1.6	26	28	0.7	0.3–1.6	11	11	0.5	0.2–1.5
Mean ± SD	3.6 ± 2.7)	3.3 ± 2.6)			2.3 ± 1.5)	2.3 ± 1.6)										
Median	3.0	2.5			2.0	2.0										
Range	1–14	1–14			1–10	1–11										

^1^Adjusted for age and sampling fractions. ^2^Adjusted for age, sampling fractions, and other covariates as specified. ^3^Additional covariates for fully adjusted OR: none. ^4^Additional covariates for fully adjusted OR: adult body mass index >25 kg/m^2^, household income ≥$30,000 and maternal age. ^5^Restricted to participants born on or after 1 January 1948. CBCS, Carolina Breast Cancer Study; CI, confidence interval; OR, odds ratio.
